# Recent Advances in the Inhibition of p38 MAPK as a Potential Strategy for the Treatment of Alzheimer’s Disease

**DOI:** 10.3390/molecules22081287

**Published:** 2017-08-02

**Authors:** Jong Kil Lee, Nam-Jung Kim

**Affiliations:** Department of Pharmacy, College of Pharmacy, Kyung Hee University, 26 Kyungheedae-ro, Dongdaemun-gu, Seoul 02447, Korea; jklee3984@khu.ac.kr

**Keywords:** p38 mitogen activated protein kinase (MAPK), kinase inhibitor, Alzheimer’s disease, tau phosphorylation, neuroinflammation, amyloid beta

## Abstract

P38 mitogen-activated protein kinase (MAPK) is a crucial target for chronic inflammatory diseases. Alzheimer’s disease (AD) is characterized by the presence of amyloid plaques and neurofibrillary tangles in the brain, as well as neurodegeneration, and there is no known cure. Recent studies on the underlying biology of AD in cellular and animal models have indicated that p38 MAPK is capable of orchestrating diverse events related to AD, such as tau phosphorylation, neurotoxicity, neuroinflammation and synaptic dysfunction. Thus, the inhibition of p38 MAPK is considered a promising strategy for the treatment of AD. In this review, we summarize recent advances in the targeting of p38 MAPK as a potential strategy for the treatment of AD and envision possibilities of p38 MAPK inhibitors as a fundamental therapeutics for AD.

## 1. Introduction

Alzheimer’s disease (AD) is an age-related, progressive, and irreversible neurodegenerative disorder characterized by cognitive and memory impairment, and is the most common cause of dementia in older adults. The estimated prevalence of this disease in 2015 was more than 40 million patients worldwide, and it is estimated that this figure will be double by 2050 [1,2,3]. Five prescription drugs are currently approved by the U.S. Food and Drug Administration (FDA) for alleviating the symptoms of AD. Three of the five available medications—donepezil, galantamine and rivastigmine—belong to a class of drugs known as “acetylcholinesterase (AchE) inhibitors”. These drugs prevent the breakdown of acetylcholine, which is important for learning and memory in the brain. The fourth drug is memantine, another class of AD drugs known as *N*-methyl-d-aspartic acid receptor (NMDAR) antagonists. Both types of drugs help attenuate symptoms but work in different ways. The fifth drug is a combination of donepezil and memantine [2]. These drugs help mask the symptoms of AD but do not cure the disease or delay its progression. Therefore, a breakthrough in AD drug development is urgently needed to treat the underlying disease and block the accompanying cell damage that eventually leads to worsening of symptoms.

**Figure 1:**
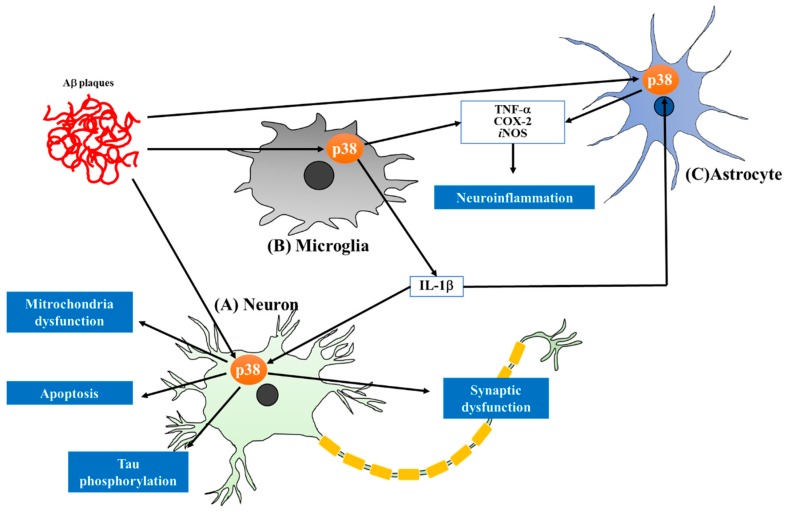
Diverse roles of p38 MAPK in AD pathologies. (**A**) Amyloid-β (Aβ) plaques evoke neuronal damages including mitochondria dysfunction, apoptosis, tau phosphorylation and synaptic dysfunction via p38 MAPK activation; (**B**) Increased microglial p38 MAPK signaling induced by Aβ is a main driver of neuroinflammation in AD, leading to production of pro-inflammatory mediators, such as interleukin-1β (IL-1β), tumor necrosis factor-α (TNF-α), cyclooxygenase-2 (COX-2) and inducible nitric oxide synthase (*i*NOS). Especially, IL-1β released from microglia stimulates p38 MAPK signaling of neuron and astrocyte in AD and exacerbates the AD brain pathology; (**C**) P38 MAPK activation in astrocyte is enhanced by Aβ plaques and IL-1β produced by microglia. This activation accelerates the neuroinflammation by releasing *i*NOS, COX-2 and TNF-α.

Protein kinases have become one of the important targets in drug discovery since the beginning of the 21st century, with marketing approvals for therapeutic applications, especially cancers [4]. However, compounds targeting protein kinases are still limited in other therapeutic areas, despite the crucial roles of these enzymes in various pathophysiological processes. Among such kinases, mitogen-activated protein kinase (MAPK) has attracted tremendous attention due to its roles in numerous cellular events, including differentiation, mitogenesis, cell survival and apoptosis [5]. P38 MAPK is a class of MAPKs responsive to stress stimuli such as inflammatory cytokines and reactive oxygen species (ROS). Many studies have revealed a central role of p38 MAPK in chronic inflammation, leading to preclinical or clinical trials for the application of p38 MAPK inhibitors in inflammatory diseases such as rheumatoid arthritis and asthma. P38 MAPK inhibitors have recently been claimed as novel and potential therapeutics for neurodegenerative diseases, a type of chronic inflammatory disease. P38 MAPK has been reported to have distinct roles in AD pathologies as activation of the p38 MAPK pathway has been observed in the postmortem brains of AD patients and animal models (Figure 1) [6,7,8]. Thus, inhibition of p38 MAPK might be a promising therapeutic strategy for the treatment of AD [9]. In this review, we summarize recent advances in the targeting of p38 MAPK as a potential strategy for the treatment of AD, focusing on the contributions of p38 MAPK pathways to AD brain pathology. In each section, we will also introduce a group of p38 MAPK inhibitors that can offer neuroprotection by mechanisms directly or indirectly involving the p38 MAPK pathways. In the section on perspectives, the current status and limitation of p38 MAPK inhibitors for AD treatment will be addressed, along with suggestions for the successful development of inhibitors for treating AD.

### 1.1. P38 Mitogen-Activated Protein Kinase (P38 MAPK) and Its Inhibitors

MAPKs are serine/threonine protein kinases that process and regulate cellular properties in response to a wide range of extracellular stimuli. These enzymes phosphorylate the OH group of serine or threonine in proteins and play important roles in the regulation of cell proliferation, differentiation, survival and apoptosis. In mammalian cells, several distinct MAPKs have been identified, including p38 MAPK, *c*-jun *N*-terminal kinase (JNK), extracellular signal-regulated kinase (ERK 1/2) and ERK 5/BMK-1. Among MAPKs, p38 MAPK is involved in a wide range of signaling pathways that stimulate different biological functions. In particular, p38 MAPK has been found to play an essential role in the regulation of pro-inflammatory signaling networks and in the biosynthesis of cytokines, including tumor necrosis factor-α (TNF-α) and interleukin-1β (IL-1β) in immune cells [10]. P38 MAPK comprises four isoforms (α, β, γ and δ). P38α and β are approximately 70% identical, whereas p38γ and δ share approximately 60% sequence identity with p38α. Among p38 isoforms, α and β are ubiquitously expressed in most tissues, including the brain [11], whereas γ and δ exhibit tissue-specific variations in expression [12,13,14,15]. Since discovering p38α is primarily responsible for regulating inflammation, most studies have intensively focused on p38α [16].

**Figure 2:**
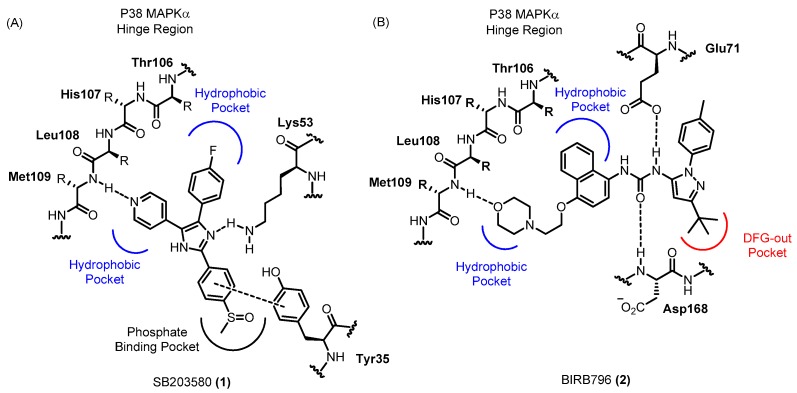
Binding modes of representative p38 MAPK inhibitors: (**A**) Binding mode of SB203580 (**1**) within p38 MAPKα; (**B**) Binding mode of BIRB796 (**2**) within p38 MAPKα.

P38α (often referred to simply as “p38”) was the first isoform of p38 MAPK to be identified and was first recognized as a stress-induced kinase that can be activated by lipopolysaccharide (LPS) and inflammatory cytokines. Many researchers have subsequently attempted to develop p38α inhibitors, which have conventionally been used to investigate the roles of p38 MAPK in the production of inflammatory cytokines leading to chronic inflammation. Inhibition of p38 MAPK has been shown to effectively alleviate inflammatory diseases such as rheumatoid arthritis, cardiovascular disease and inflammatory pain [12,17,18,19]. Thus, p38 MAPK inhibitors are considered novel potential drug candidates for inflammation-related diseases. Since the identification of the prototypical p38 MAPK inhibitor **1** in 1994, numerous p38 MAPK inhibitors have been developed and have served as efficient tools for further understanding the roles of this kinase [20,21]. P38 MAPK inhibitors vary markedly in both chemical structure and binding mode [22]. The binding modes of the representative p38 MAPK inhibitors, **1** and **2** are illustrated in Figure 2. In the binding mode of **1** within p38 MAPKα, the hydrogen bonding between Met109 and *N* of pyridine moiety is known as a crucial interaction. Another hydrogen bonding and additional hydrophobic interactions have been known to be beneficial. Most p38 MAPK inhibitors, including **1**, are adenosine triphosphate (ATP)-competitive and bind to the hinge region in p38 MAPK. Other inhibitors, including **2**, do not compete with ATP for the adenosine binding pocket but evoke a conformational reorganization of p38 MAPK that prevents ATP binding [23]. Especially, **2** binds “DFG-out” conformation of p38 MAPKα, which is not catalytically active because of its low binding affinity to ATP. Many p38 MAPK inhibitors have progressed to clinical trials, but in most cases, the clinical trials have been withdrawn because of side effects in the liver and central nervous system (CNS), partly due to off-target effects. Cross-reactivity with other kinases might underlie these side effects. In addition, the efficacy of the drugs in clinical trials was not sufficient for the target diseases. To overcome these limitations, novel inhibitors with high kinase selectivity profiles are necessary, as well as careful selection of disease models and clinical applications.

In the CNS, p38 MAPK is highly expressed in regions that are crucial for learning and memory and is likely a key component in higher brain functions [11]. Therefore, dysfunction of this pathway might be related to the pathology of some neurological disorders, such as AD, ischemia, neuropathic pain, epilepsy and depression [11]. P38 MAPK has also been implicated in inhibition of embryonic stem cell differentiation into neurons, regulation of synaptic plasticity and modulation of neuronal excitability [24,25,26]. Inhibition of p38 MAPK has been assessed in in vivo and in vitro experiments using various models of neurological disorders, and, in most cases, these p38 MAPK inhibitors have been shown to be effective, indicating that they could be used for the treatment of neurological disorders including AD [11,27,28,29].

### 1.2. Alzheimer’s Disease (AD) and Recent Drug Candidates Targeting AD Pathologies

**Figure 3:**
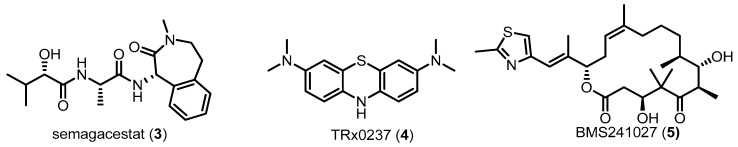
Examples of novel compounds targeting Aβ and tau pathologies.

AD is clinically characterized by a progressive loss of cognitive function including memory, language, calculations, orientation and judgment [30,31]. Thus, AD is a type of neurodegenerative disease. It is increasingly clear that AD, like most other neurodegenerative diseases, is fundamentally related to alterations of protein folding and aggregation. AD is a multifactorial disorder. To clarify causative factors associated with AD, several hypotheses have been proposed, including the “amyloid-β (Aβ) hypothesis”, “tau hypothesis”, “cholinergic hypothesis”, and “neuroinflammation hypothesis” [32]. The two primary neuropathological hallmarks of AD are extracellular senile plaques and intraneuronal neurofibrillary tangles (NFTs) in regions of the brain such as the hippocampus and cortex [33]. It has been known for decades that AD-related mutations in amyloid precursor protein (APP) and presenilins (PS) 1 and 2 increase the levels of Aβ peptide [34]. APP is an integral membrane protein that is sequentially cleaved by α-, β- and γ-secretase to produce Aβ40 and Aβ42. Aggregated Aβ peptides are associated with progressive neuronal degeneration in AD. More recent studies have shown that additional Aβ peptides, such as Aβ43, Aβ45, Aβ48, and Aβ49, are also found in AD patients [35]. Aβ43 appears to be more toxic than Aβ42, and longer Aβ peptides are highly self-aggregating [35,36]. Because Aβ is highly toxic to neuronal cells, extensive investigations have focused on the inhibition of Aβ production and accumulation as an effort to develop AD therapeutics. For example, a series of drug candidates targeting Aβ processing, including the γ-secretase inhibitor **3**, have been developed and evaluated in clinical trials for AD patients (Figure 3) [37,38,39]. Although these drugs had therapeutic effects in AD animal models, they had low efficacies or side effects in AD patients.

NFTs are intracellular aggregates of filamentous forms of the microtubule-associated protein tau. In healthy individuals, tau is predominantly localized in the axons of neurons and functions to promote microtubule assembly, stability, nucleation and vesicle transport [40]. Braak et al. first reported that the spatial and temporal patterns of tangles in the brains of AD patients are associated with dysfunction of neuronal networks and are positively correlated with cognitive decline. In AD, NFTs first appear in the transentorhinal cortex and spread to the hippocampus and, eventually, the cortex [41]. In AD, tau becomes hyperphosphorylated, leading to microtubule destabilization, thereby reducing axonal transport ability. In addition, axonal swellings, or varicosities, which are frequently observed in early-stage AD, are related with tau-associated defects in the transport of cargo-containing vesicles [42]. Some evidences indicate that tau also regulates synaptic function, and it is found in the pre- and postsynaptic components of neurons. Therefore, synaptic decline in AD might be related to hyperphosphorylated tau [43]. A series of kinases that phosphorylate tau in the brains of AD patients have been identified (e.g., glycogen synthase kinase 3, cyclin-dependent kinase, casein kinase 1, and p38 MAPK). Consequently, numerous therapeutic approaches targeting tau have been addressed, including active and passive tau immunotherapy, tau-lowering drugs and kinase inhibitors for inhibition of tau hyperphosphorylation. Some of the drug candidates targeting tau, such as **4** and **5**, have been tested in clinical trials to demonstrate therapeutic efficacy in AD (Figure 3) [44].

Neuroinflammation is a type of immune response in the CNS and is observed in diverse neurodegenerative disorders, such as AD, depression, multiple sclerosis and Parkinson’s disease. Accumulating evidence indicates that neuroinflammatory processes also directly contribute to AD pathogenesis, including the activation of astrocytes and microglia, which can increase the expression of cytokines or chemokines [45]. Microglia, known as macrophages of the CNS, play pivotal roles in neuroinflammatory responses and in their activated form, release inflammatory factors in response to inflammatory mediators such as toxic molecules and cytokines. Astrocytes, the most abundant cells in the CNS, also release inflammatory signaling molecules and have key roles in synaptic regulation and function. Aβ peptides and hyperphosphorylated tau engage in cross-talk with neuronal cells and glia via pro-inflammatory cytokines [46]. The association between AD and inflammatory processes has also been clinically proven. Elevated levels of IL-1 and TNF-α are related to the progression of AD [47]. Nitric oxide (NO), a representative inflammatory mediator, is enhanced in AD and is related to Aβ deposition and disease progression [48]. Production of Aβ42 by neurons evokes cascades of events comprising mitochondrial dysfunction, resulting in oxidative stress, tau hyperphosphorylation, caspase 3 enzyme activation, and overproduction of ROS and NO via damage of the astrocyte-neuron contact, leading to the destruction of neuronal synapses [49]. Taken together, these findings indicate that alleviation of neuroinflammation could be an effective approach for the treatment of AD. Therefore, several studies have attempted to show beneficial effects of anti-inflammatory drugs, especially nonsteroidal anti-inflammatory drugs (NSAIDs) such as naproxen and diclofenac, for AD, but the trials failed to show beneficial effects [50,51,52,53]. This failure might be due to the treatment dosage, timing and specificity of the drugs. Although the trials were not successful, diverse clinical, genetic, and basic science data support the importance of neuroinflammation in the progression of AD. Consequently, many research groups have sought to demonstrate the possibilities of anti-inflammatory drugs as part of a new therapeutic strategy for AD.

## 2. Recent Advances in the Study of p38 MAPK and Its Inhibition in AD Pathology

In 1999, Hensley et al. reported that p38 MAPK activity was increased in the brains of AD patients [54]. In the human AD brain, p38 MAPK activation occurs in the early stage, as confirmed by studies using human post-mortem tissues from control and Alzheimer’s cases [6,55]. In addition, MAPK kinase 6 (MKK6), an upstream activator of p38 MAPK, is upregulated in AD post-mortem brains [56]. A recent clinical study reported that phosphorylated p38 MAPK in the blood of AD patients is positively correlated with disease duration [57]. Taken together, these findings indicate that the p38 MAPK pathway is associated with the pathogenesis of AD. Therefore, several research groups have attempted to investigate the role of this kinase in AD pathologies, such as NFTs in neurons and Aβ plaques. In addition, because neuroinflammation is a representative symptom of AD, inhibition of p38 MAPK, which is known to significantly alleviate inflammatory diseases by decreasing pro-inflammatory cytokines, is a promising target. In this section, we briefly summarize studies of the inhibition of p38 MAPK by small-molecule inhibitors to alleviate the pathologies of AD, with a focus on recent findings.

### 2.1. Targeting the Tau Pathology via p38 Inhibition in Neuronal Cells

**Figure 4:**
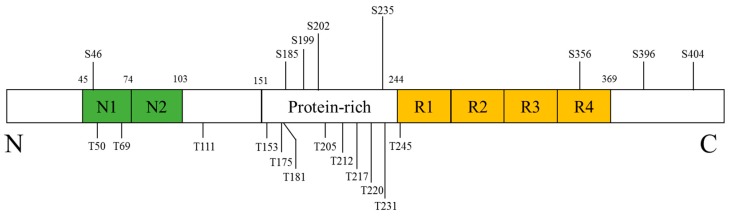
Schematic representation illustrating the tau phosphorylation sites by p38 MAPK.

Tau, in its longest isoform, is a phosphoprotein with 45 serine, 35 threonine, and 5 tyrosine residues meaning that nearly 20% of the tau protein has the potential to be phosphorylated. In normal condition, tau is promoting microtubules assembly, stabilizing microtubules and involving in neurite outgrowth [58,59]. However, high degrees of tau phosphorylation are observed in the AD, which have been known as a tauopathy implicated in AD pathology. The NFTs, a kind of main pathological structures of AD, are made up of paired helical filaments featured with hyperphosphorylated tau [60]. The accumulation of these abnormal proteins results in the microtubule dysfunction and abnormal axonal transport, and eventually impairs neural function [61]. Numerous kinases, including more than 20 serine/threonine kinases, were suggested to phosphorylate of tau [62,63,64]. Several kinases that are candidates for disease-related tau phosphorylation include glycogen synthase kinase-3, cyclin-dependent kinase-5 and the MAPK family, all of which have been known to be implicated in AD pathogenesis [64]. Among them, p38 MAPK has been suggested to phosphorylate tau at specific amino acid residues (Figure 4) [65,66,67,68,69].

**Figure 5:**
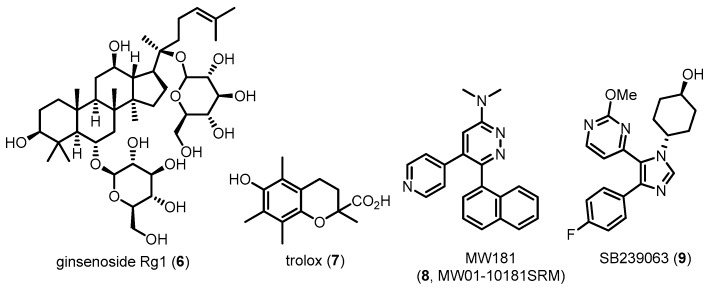
Structures of the compounds known to inhibit tau phosphorylation by modulating the p38 MAPK pathway.

In vivo studies using transgenic mice featured with hyperphosphorylated tau have demonstrated that p38 MAPK activation is positively correlated with the amount of aggregated tau [70]. P38 MAPK is exclusively localized and associated with neurofibrillar pathology in hippocampal and cortical brain regions of postmortem samples from AD patients [71]. In particular, tau protein in neuronal cells is phosphorylated via p38 MAPK cascades induced by inflammatory cytokines such as IL-1 or innate immune cells, including microglia and astrocytes [72,73,74]. Furthermore, activated p38 MAPK in neuronal cells is co-localized with hyperphosphorylated tau as well as activated microglia overexpressing IL-1β [65]. These results indicate that p38 MAPK is significantly associated with tau phosphorylation and that its inhibitors could potentially suppress this phosphorylation, leading to alleviation of tau pathologies in AD. Therefore, several groups have attempted to decrease tau pathologies related to tau phosphorylation using direct or indirect p38 MAPK inhibitors (Figure 5). Since the role of p38 MAPK in tau protein phosphorylation was revealed, commercially available p38 MAPK inhibitors such as **1** have been conventionally used as reference tools for biological evaluation. Other p38 MAPK inhibitors have also been evaluated in AD models. Compound **6** was reported to attenuate tau hyperphosphorylation in Aβ-stimulated neuronal cells via inhibition of p38 activation [75]. Recently, a water-soluble vitamin E analog **7** was reported to decrease tau toxicities in neuronal cells by inhibiting oxidative stress-induced p38 activation [76]. Liu et al. reported that proanthocyanidins, a class of naturally occurring flavonoids, inhibit ER stress-induced p38 activation cascades, leading to a decrease in the amount of phosphorylated tau and improvement of lead-induced cognitive impairments [77]. These compounds do not directly inhibit p38 MAPK but might target relevant signaling or upstream pathways of p38 MAPK. More recently, Bhaskar’s group reported that a recently reported selective p38α MAPK direct inhibitor **8** [78] suppresses p38α MAPK activation, leading to reduced tau phosphorylation and preventing cognitive impairment in aged hTau mice. Another p38α MAPK inhibitor **9** was used as a positive control. As shown above, most studies have used indirect p38 MAPK inhibitors to alleviate tau pathology. Further in vitro/vivo studies using direct inhibitors are necessary for a detailed investigation of tau phosphorylation related to AD pathology.

### 2.2. Neuroprotective Effect of p38 Inhibition against Aβ-Induced Neuronal Damage

**Figure 6:**
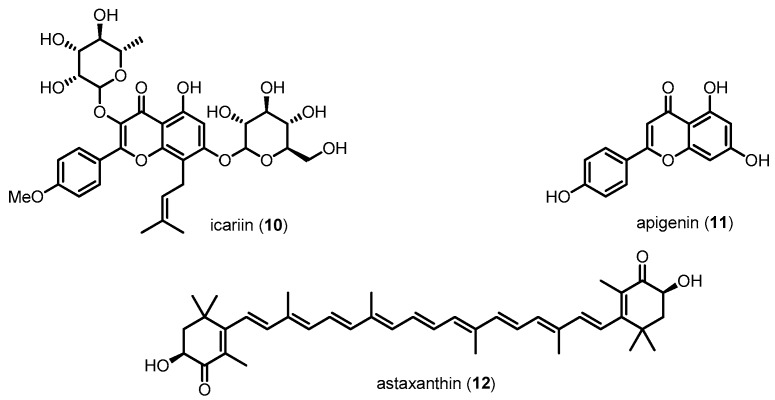
Natural compounds reducing Aβ-induced neurotoxicity via p38 MAPK inhibition.

Aβ, a pathological hallmark of AD, causes neuronal damage via oxidative stress, caspase activation and mitochondrial dysfunction. Toxic Aβ, including Aβ40 and Aβ42, is generated from the cleavage of APP by β-site APP-cleaving enzyme 1 (BACE1) and γ-secretase complex. The resultant Aβ sequentially aggregates into oligomers and forms fibrils that are deposited in diffuse or compact plaques. The original hypothesis for Aβ toxicity was that extracellular deposits of aggregated Aβ were responsible for neuronal damage. Mounting evidence indicates that intraneuronal accumulation of Aβ is also a pathological event in AD [79,80,81]. A number of studies have revealed a significant role of p38 MAPK related to Aβ in neuronal cells of AD. Activation of p38 MAPK by Aβ results in increased intracellular calcium, ROS production/accumulation, and mitochondrial stress, all of which have been implicated in the pathology of AD neurons [82,83]. In addition, p38 MAPK activation upregulated its direct downstream target, *c*-Jun, known as an apoptotic transcription factor that can cause neuronal cell death in AD [84]. In vitro studies using primary cultured neurons and immortalized neuronal cells have shown that exposure to Aβ in these cells activates p38 MAPK and contributes to neuronal apoptosis [85,86,87,88]. These results were further confirmed in various AD animal models [89,90,91,92,93]. Recent works have reported that Aβ-induced p38 MAPK phosphorylation increases ROS, leading to neuronal cell death [94,95]. It has been also reported that Aβ-triggered microglial release of inflammatory mediators, especially IL-1β, activates neuronal p38 MAPK cascades [72,96]. These evidences of the involvement of p38 MAPK in Aβ-induced neuronal damage imply that p38 MAPK activity plays a significant role in the progression of the clinical signs and pathology of AD. Based on these results, many groups have attempted to inhibit the p38 MAPK pathway in AD neuronal cells. Researchers are also trying to improve knowledge of the exact roles of p38 MAPK in AD neurons (Figure 6). For example, **10**, **11** and **12**, a class of natural compounds, reduce Aβ-induced neurotoxicity and neuronal apoptosis via inhibition of p38 MAPK in a dose-dependent manner [97,98,99].

**Figure 7:**
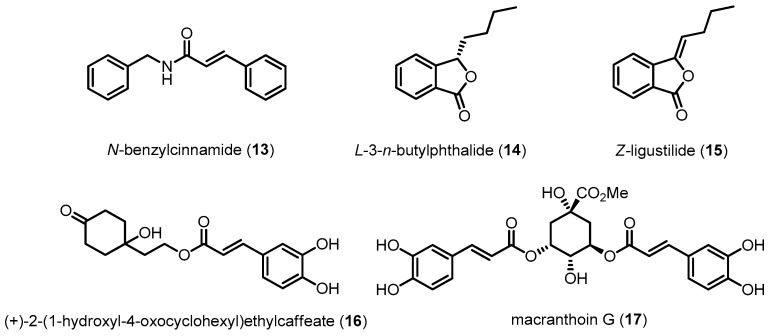
Compounds reducing Aβ-induced neurotoxicity via p38 MAPK inhibition I.

Other compounds, such as **13**, **14**, **15**, **16** and **17**, also have protective effects against the cytotoxicity induced by Aβ or hydrogen peroxide in neuronal cells (Figure 7). The neuroprotective effects of these compounds are related to the downregulation of p38 MAPK [100,101,102,103,104].

**Figure 8:**
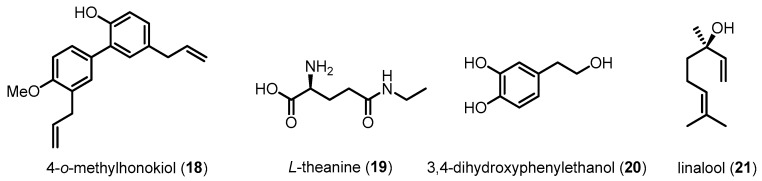
Compounds reducing Aβ-induced neurotoxicity via p38 MAPK inhibition II.

**Figure 9:**
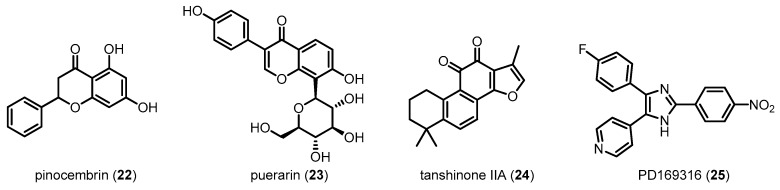
Compounds reducing Aβ-induced neurotoxicity via p38 MAPK inhibition III.

The inhibitory effects of p38 MAPK on Aβ-induced neuronal damages, including memory impairment, neuronal apoptosis and mitochondrial dysfunction, have been further examined using several small molecules in AD animal models (Figure 8 and Figure 9). For example, **18** extracted from Magnolia officinalis and **19** derived from green tea prevent memory impairment and neuronal cell death by reducing p38 MAPK activation in an Aβ42-infused AD mouse model [105,106]. Treatment of **20** effectively inhibits oligomeric Aβ42-evoked phosphorylation of p38 MAPK in C57BL/6 mice [107]. Compound **7**-treated APP/PS1 double transgenic mice exhibit a reduction of p38 MAPK activation and oxidative stress in the hippocampus [76]. Similarly, **21**-treated 3xTg-AD mice show improvements in learning and spatial memory [108].

In Aβ-induced AD mice, oral administration of **22**, a flavanone abundant in propolis, improves behavioral performance and restores neuronal function by inhibiting the p38 MAPK pathway. In addition, the mice significantly exhibited alleviation of the mitochondrial dysfunction via improved mitochondrial membrane potential and inhibition of mitochondrial oxidative stress [109]. The positive effects of p38 MAPK inhibition against mitochondrial dysfunction were further confirmed in mitochondrial transgenic neuronal cell hybrid sporadic AD models after the treatment of **23** [110]. Protective effects of **24** against memory deficits and neuronal dysfunction were observed in streptozotocin-induced sporadic AD mice [111]. Interestingly, partial genetic deletion of p38 MAPK in neurons reduced Aβ generation and decreased Aβ plaques by promoting autophagy-associated BACE1 degradation [8]. More importantly, recent studies using direct p38 MAPK inhibitors, such as **9** and **25**, have demonstrated that inhibition of p38 MAPK significantly decreases neuronal loss as well as inflammation and oxidative stress in cerebral ischemia-induced APP transgenic [112] and Aβ-infused AD mice [28]. Although reduction of Aβ damages by p38 MAPK inhibitors is no doubt a promising therapeutic strategy for AD, the exact mechanisms of direct p38 MAPK inhibitors and further development are needed for clinical application.

### 2.3. Reduction of Neuroinflammation by Inhibiting p38MAPK Pathway in Microglia and Astrocytes

**Figure 10:**
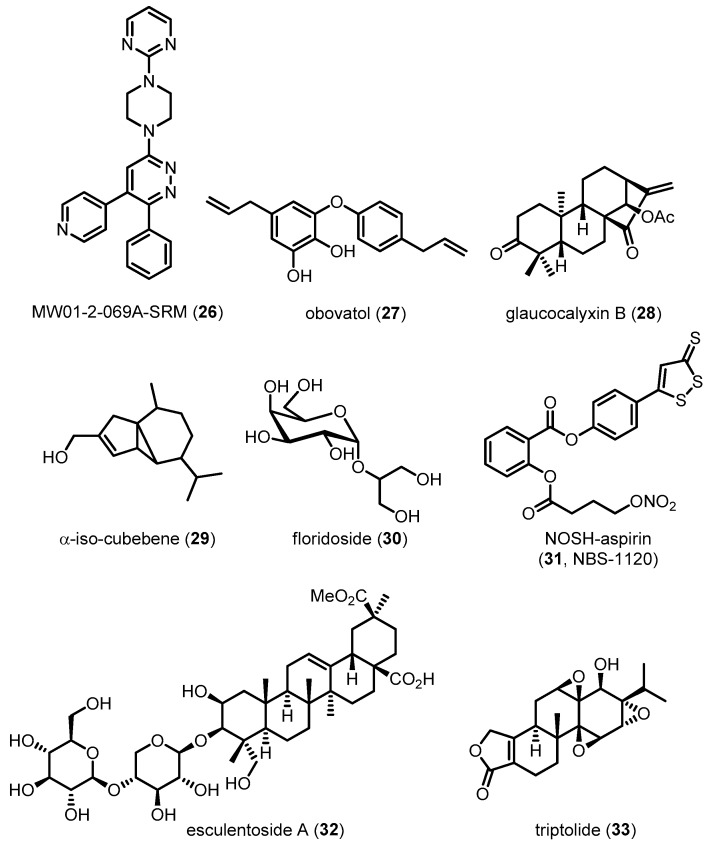
Structures of compounds known to suppress inflammation in glia by modulating the p38 MAPK pathway.

In addition to pathological markers of disease such as Aβ and NFTs, neuroinflammation is an important symptom of AD. The brain is mainly defended by the innate immune response involving activated glia such as microglia and astrocytes, which can produce key inflammatory mediators. The role of microglia in AD pathogenesis has been debated for past decades. For example, microglia involve the Aβ clearance and survival of neurons [113,114], whereas persistent microglial pro-inflammatory activation accelerates amyloidosis, neuronal damage and neuroinflammation [115]. Under normal conditions, phenotype of microglia is regulated by neurons and astrocytes for maintaining their phagocytic activity and keeping the normal brain microenvironment [116,117]. In the case of AD progress, however, microglia lose the regulatory function and become more sensitive to inflammatory stimuli [118]. They exacerbate Aβ accumulation and neuronal loss, resulting in a cycle of microglial priming and release of pro-inflammatory cytokines, with a subsequent elevation of AD pathology [118,119]. In addition, astrocytes have been considered to have opposite roles in the normal and AD conditions, respectively. For example, astrocytes can modulate Aβ-mediated neurotoxicity, and remove Aβ, leading to the creation of a protective barrier that surrounds plaques [120,121]. However, excessive cellular stress caused by Aβ might induce the abnormal activation of astrocytes, which make the cells release pro-inflammatory mediators including TNF-α, IL-1β and nitric oxide, leading to a state of chronic inflammation in the AD brain. Activated microglia and astrocytes were found near Aβ and correlated with NTF in AD, indicating the significant correlation between neuroinflammation and AD pathology. Therefore, modulation of neuroinflammation is necessary for the alleviation of AD progression. The roles of the p38 MAPK pathway in glial cells have been studied in recent decades. In 1998, Bhat et al. reported that p38 MAPK cascades contribute to transcriptional and post-translational regulation of inducible nitric oxide synthase (*i*NOS) and TNF-α gene expression in LPS-activated glial cells [122]. This was one of the first examples of the pro-inflammatory role of p38 MAPK in glial cells, which was further observed in human microglia and astrocytes [123]. LPS-stimulated p38 MAPK cascades in murine cells have been associated with the production of IL-1β [124]. Interestingly, Aβ fibril, a representative pathological marker in AD, has also been identified as a stimulus activating p38 MAPK cascades for the production and upregulation of pro-inflammatory cytokines in microglia [125]. Giovanni et al. validated these findings in in vivo experiments by showing that p38 MAPK signal transduction in microglial cells is crucial for Aβ-induced neuroinflammation [126]. Astrocytes, another type of glial cells, are also associated with neuroinflammation. IL-1β released from microglia affects predominantly astrocytes by activation of nuclear factor kappa-light-chain-enhancer of activated B cells (NF-kB) cascades [127]. Because IL-1β can activate p38 MAPK pathways in human and rat astrocytes, p38 MAPK in these cells has been assumed to play a role in the exacerbation of neuroinflammation [123,128,129]. P38 MAPK in astrocytes has also been reported to be involved in astrocytic *i*NOS and TNF-α production via direct transcriptional control, resulting in chronic neuroinflammation [123,130]. Recently, Saha et al. demonstrated that p38 MAPK can suppress the increase in the transcriptional activity of the transcription factor NF-kB mediated by IL-1β in primary human astrocytes [131]. These results imply that p38 MAPK is significantly involved in glial activation and subsequent neuroinflammation, leading to chronic neurotoxicity. Therefore, several groups have attempted to block p38 MAPK signaling in glial cells for the alleviation of AD (Figure 10). For example, Munoz et al. demonstrated that, in brain inflammation, activated p38 MAPK is significantly inhibited by **26**, a direct p38α MAPK inhibitor, leading to the alleviation of neurotoxicity [132]. Natural compounds such as **27** and **28** have been reported to decrease glia-mediated neuroinflammation in microglia and astrocytes via a mechanism involving inactivation of the p38 MAPK signaling pathway [133,134]. Compound **29** also exhibits anti-inflammatory activities via inhibition of p38 MAPK activation in Aβ-stimulated microglia [135]. A sugar analog **30** suppresses pro-inflammatory responses in microglia and down-regulates *i*NOS and cyclooxygenase-2 (COX-2) by blocking p38 MAPK phosphorylation in LPS-treated BV-2 cells [136]. Recently, **31**, a novel hybrid of aspirin and chemical moieties that release NO and hydrogen sulfide (H_2_S), was found to attenuate activation of p38 MAPK, leading to alleviation of neuroinflammation in cells [137]. Interestingly, donepezil, a well-known AchE inhibitor, exhibits anti-inflammatory activities and inhibits microglial activation in vitro and in vivo. Oh et al. suggested that donepezil might also modulate p38 MAPK in addition to inhibiting AchE, thereby contributing to the amelioration of neurodegeneration [138]. Compound **32** inactivates p38 MAPK stimulated by Aβ in the hippocampus of AD mice, suggesting that it can suppress the Aβ-induced activation of glia [139]. Similar phenotypes with decreased histopathological hallmarks of AD via anti-inflammatory effects have been observed in response to a natural monoterpene **21** [108]. More recently, **33** was found to suppress the activation of inflammatory pathways such as p38 MAPK in glial cells, leading to the alleviation of neurotoxicity in AD-induced mice [94].

As shown above, most studies have focused on the suppression of neuroinflammation in activated glial cells via indirect p38 MAPK inhibitors such as natural terpenoids. Therefore, additional studies using direct p38 MAPK inhibitors are necessary to further understand the underlying pathology of neuroinflammation in AD. In particular, considering that most of the studies of Aβ pathologies mentioned in Section 2.2 have indicated that Aβ-induced toxicities in neuronal cells are also associated with p38 MAPK signaling in these cells, intensive molecular biology studies and additional in vivo studies might be necessary to understand the cross-talk between neuroinflammation by activated glia and neuronal toxicity via p38 MAPK signaling in terms of systemic AD pathologies.

### 2.4. Improvement of Synaptic Plasticity by p38 Inhibition

The hippocampus is an area of the brain that is required for the formation of learning and memory. This type of formation is significantly regulated by synaptic plasticity and includes long-term increases in synaptic strength, termed long-term potentiation (LTP), and long-term decreases in synaptic strength, referred to as long-term depression (LTD). The process can be induced by the activation of NMDAR or metabotropic glutamate receptors (mGluRs). Many studies have reported that Aβ-induced impairments in synaptic plasticity coincide with memory decline [140,141]. P38 MAPK has been recognized as a signal transducer in mGluR- and NMDAR-dependent LTD formation in the hippocampus [142,143,144], whereas p42/p44 MAPK functions in LTP [142]. Despite the importance of the relationship between synaptic plasticity and p38 MAPK, knowledge in AD is limited. The importance of mGluR-LTD in AD is supported by findings that inhibition of mGluR5 recovers memory impairment and reduces Aβ plaque load in AD mice [145,146]. Aβ enhances LTD in the hippocampal dentate gyrus region, and Aβ facilitates LTD involving mGluR1/5, p38 MAPK and caspase-3 activation [147]. In addition, the elevation of Aβ in cultured hippocampal slices induces mGluR-LTD in a p38 MAPK-dependent manner by promoting the phosphorylation and endocytosis of α-amino-3-hydroxy-5-methyl-4-isoxazolepropionic acid receptors (AMPARs), which triggers the dendritic spine loss that evokes memory dysfunction in AD [148]. The Aβ-mediated internalization of AMPARs might contribute the loss of synapses observed in AD [149]. Ashabi et al. showed that a p38 MAPK inhibitor **22** reduces cognitive impairments in AD [28]. Although further evaluation is necessary to delineate the exact mechanism, the positive effects of the inhibition of p38 MAPK might be caused by suppressing the endocytosis of AMPARs in response to exposure to endogenous Aβ and thereby rescuing the damage to LTP [150]. Several recent experiments have sought to confirm the relationship between p38 inhibition and synaptic improvement in AD. In Aβ-treated cells, LTP was significantly blocked, and LTD was dramatically facilitated. However, these effects were prevented by a direct p38 MAPK inhibitor **1** [141,150,151]. Given these results, targeting p38 MAPK could be useful for ameliorating synaptic dysfunction and recovering synaptic plasticity, although additional studies are necessary to reveal the exact mechanism.

As mentioned above, activation of p38 MAPK is significantly associated with tau phosphorylation. Hyperphosphorylated tau might have a role in regulating important processes related to synaptic function as well as axonal transport in AD [43]. Abnormal axonal transport by hyperphosphorylated tau might affect a number of presynaptic mitochondria, thus impairing release of synaptic vesicles. Tau could also act as a protein scaffold, and regulation of its binding partners might alter signaling pathways. In the postsynapse, tau interacts with the postsynaptic density protein-95/NMDAR complex. In AD, abnormal tau might accelerate overactivation of NMDARs, resulting in toxic effects on neurons [152,153]. Recent studies have indicated that tau is involved in LTD in CA1 of the hippocampus [154,155]. Although direct evidence linking p38 MAPK inhibitors and restoration of synaptic function in AD is somewhat lacking, many studies have suggested that normalization of tau function by inhibition of p38 MAPK could be a therapeutic approach to recover the synaptic dysfunction observed in AD.

## 3. Conclusions and Perspectives

**Figure 11:**
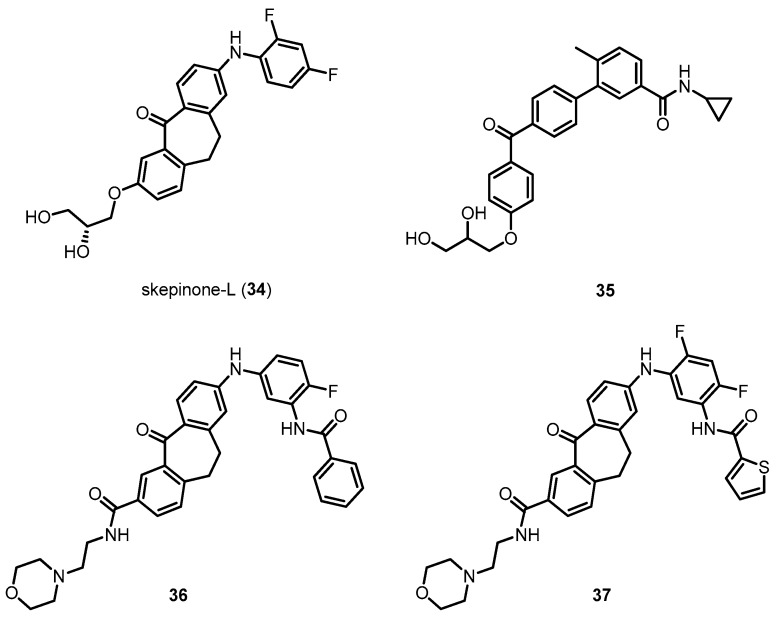
Examples of recently reported potent and selective p38 MAPK inhibitors.

Emerging results outlined in this review and other reports omitted due to limited space clearly indicate that p38 MAPK has a distinct role in AD pathophysiology (Table 1). The localization and activation of p38 MAPK are positively correlated with tau phosphorylation, which is significantly driven by neuroinflammation by activated microglia and astrocytes. In addition, p38 MAPK is a crucial enzyme in the process of Aβ-mediated neuronal toxicities. Aβ and phosphorylated tau, both important AD pathologies, as well as neuroinflammation and synaptic plasticity are significantly alleviated by the direct or indirect inhibition of p38 MAPK, indicating that p38 MAPK inhibitors would be very promising drug candidates for AD treatment. Although the recent studies have elucidated the roles of p38 MAPK in neurodegenerative disease, detailed investigations for the clinical usefulness of direct p38 MAPK inhibitors for AD have not intensively progressed. Most studies using p38 MAPK inhibitors have focused on chronic inflammatory diseases such as rheumatoid arthritis and asthma. Therefore, focused efforts to use p38 MAPK inhibitors for challenging diseases such as AD are urgently needed. The development of p38 MAPK inhibitors for clinical use in AD remains challenging. To succeed, several points should be considered and investigated. First, detailed mechanistic studies of AD should be conducted using potent and selective p38 MAPK inhibitors. Most studies of p38 MAPK in AD pathologies have been investigated using indirect inhibitors. In addition, most studies using direct p38 MAPK inhibitors have used SB203580 (**5**) and other commercially available inhibitors with lower potency and selectivity than recently reported p38 MAPK inhibitors such as **34** and compound **35** (Figure 11) [156,157,158,159,160,161]. In particular, a series of p38 MAPK inhibitors, **34** and **35**, possessing diaryl ketone moieties have been reported to make potent bindings with the p38 MAPK, due to their carbonyl oxygens which can induce glycine flip, leading to double hydrogen bonding to Met109 and Gly110 in the hinge region. Glycine flip would occur in the case of glycine directly adjacent to the linker residue such as methionine, and this arrangement exists in 9.2% of all kinases, indicating that the flip interaction can make tight and selective bonding of the compounds toward the kinase. Therefore, these compounds exhibited remarkable kinase selectivity profiles. More recently, novel p38 MAPK inhibitors including **36** and **37** have been reported to target both active and inactive states of p38 MAPK for the increased target residence time. They are made up of glycine flip-inducing moieties and binders interacting with the R-spine, which make the compounds have excellent kinase selectivities, significantly potent activities on the enzyme level and in the cell based assay (Figure 11) [162]. The use of potent and selective p38 MAPK inhibitors would further reveal their potential for the treatment of AD and reduced off-target effects. Along with this work, novel p38 MAPK inhibitors with improved selectivity and pharmacokinetic profiles should be developed via rational design.

Second, the biology underlying AD pathology should be further investigated, with a specific focus on the subtypes of p38 MAPK. Tau phosphorylation does not always result in AD pathogenesis. Depending on the subtype of p38 MAPK, inhibition may cause progression of AD. Ittner et al. recently showed that p38γ-mediated site-specific phosphorylation of tau inhibits amyloid-β toxicity in an animal model [164]. In addition, p38α-specific inhibition was effective in memory recovery and ameliorating AD symptoms in in vitro/vivo models [163]. Therefore, intensive studies of the relationship between p38 MAPK subtypes and tau pathology are needed. Third, novel p38 MAPK inhibitors permeable to the blood-brain barrier (BBB) should be developed to avoid efflux from the inner area of the brain. Small molecules with a molecular weight of less than 500 can pass through the BBB. However, relatively lipophilic compounds are reportedly effluxed from the CNS by p-glycoprotein and other mechanisms [165]. Therefore, the physicochemical properties of these compounds must be optimized. Finally, the safety of p38 MAPK inhibitors is an important concern. P38 MAPK, especially p38α, belongs to a class of abundant kinases in the human body that has various physiological roles in cellular survival and sustainability. Thus, the inhibition of p38 MAPK might result in toxicity to normal human cells. Therefore, careful monitoring to determine the optimal therapeutic index (TI; CC_50_/EC_50_) is necessary. Nevertheless, p38 MAPK continues to emerge as a promising drug target for AD, and its inhibitors hopefully represent potential therapeutic strategies for AD and related neurodegenerative diseases.

## Figures and Tables

**Table 1:** Compounds alleviating Alzheimer’s disease via modulation of p38 MAPK pathway.

Compound	M.W. ^1^	Mode of Action	Activities	Reference
Ginsenoside Rg1 (**6**)	801.01	Inhibiting p38 MAPK activation	Attenuating tau hyperphosphorylation	[75]
Trolox (**7**)	250.29	Inhibiting p38 MAPK activation	Decreasing tau toxicities	[76]
MW181 (**8**)	326.39	Directly inhibiting p38 MAPK	Attenuating tau hyperphosphorylation/Preventing cognitive impairments	[78,163]
SB239063 (**9**)	368.40	Directly inhibiting p38 MAPK	Attenuating tau hyperphosphorylation/Preventing cognitive impairments	[78]
Icarin (**10**)	676.66	Inhibiting p38 MAPK activation	Reducing Aβ-induced neurotoxicity	[97]
Apigenin (**11**)	270.24	Inhibiting p38 MAPK activation	Reducing Aβ-induced neurotoxicity	[99]
Astaxanthin (**12**)	596.84	Inhibiting p38 MAPK activation	Reducing Aβ-induced neurotoxicity	[98]
*N*-Benzylcinnamide (**13**)	237.30	Inhibiting p38 MAPK activation	Reducing Aβ-induced neurotoxicity	[101]
l*-*3-*n*-Butylphthalide (**14**)	190.24	Inhibiting p38 MAPK activation	Reducing Aβ-induced neurotoxicity	[102]
*z*-Ligustilide (**15**)	188.22	Inhibiting p38 MAPK activation	Reducing Aβ-induced neurotoxicity	[104]
(+)-2-(1-Hydroxyl-4-oxocyclohexyl) ethylcaffeate (**16**)	320.34	Inhibiting p38 MAPK activation	Reducing H_2_O_2_-induced neurotoxicity	[103]
Macranthoin G (**17**)	530.48	Inhibiting p38 MAPK activation	Reducing H_2_O_2_-induced neurotoxicity	[100]
4-*O*-methylhonokiol (**18**)	280.36	Inhibiting p38 MAPK activation	Reducing Aβ-induced neurotoxicity and neuroinflammation/Preventing memory impairment	[105]
l-Theanine (**19**)	160.17	Inhibiting p38 MAPK activation	Reducing Aβ-induced neurotoxicity/Preventing memory impairment	[106]
3,4-Dihydroxyphenyl ethanol (**20**)	154.16	Inhibiting p38 MAPK activation	Reducing Aβ-induced neurotoxicity/Preventing memory impairment	[107]
Linalool (**21**)	154.25	Inhibiting p38 MAPK activation	Attenuating tau hyperphosphorylation/Improving learning and spatial memory/Reducing neuroinflammation	[108]
Pinocembrin (**22**)	256.25	Inhibiting p38 MAPK	Reducing Aβ-induced neurotoxicity/Improving behavioral performance	[109]
Puerarin (**23**)	416.38	Inhibiting p38 MAPK	Alleviating mitochondrial dysfunction	[110]
Tanshinone IIA (**24**)	294.34	Inhibiting p38 MAPK	Preventing memory impairment	[111]
PD169316 (**25**)	360.34	Directly inhibiting p38 MAPK	Reducing Aβ-induced neurotoxicity	[28]
MW01-2-069A-SRM (**26**)	395.46	Directly inhibiting p38 MAPK	Reducing neuroinflammation/Improving behavioral performance	[132]
Obovatol (**27**)	282.33	Inhibiting p38 MAPK activation	Reducing neuroinflammation	[133]
Glaucocalyxin B (**28**)	358.47	Inhibiting p38 MAPK activation	Reducing neuroinflammation	[134]
α-iso-cubebene (**29**)	220.35	Inhibiting p38 MAPK activation	Reducing neuroinflammation	[135]
Floridoside (**30**)	254.23	Inhibiting p38 MAPK activation	Reducing neuroinflammation	[136]
NOSH-aspirin (**31**, NBS-1120)	461.47	Inhibiting p38 MAPK activation	Reducing neuroinflammation	[137]
Esculentoside A (**32**)	826.96	Inhibiting p38 MAPK activation	Reducing neuroinflammation/Improving learning and spatial memory	[139]
Triptolide (**33**)	360.40	Inhibiting p38 MAPK activation	Reducing neuroinflammation/Improving learning and spatial memory	[91]
Skepinone-l (**34**)	425.42	Directly inhibiting p38 MAPK	ND ^2^	[156]
Compound **35**	445.51	Directly inhibiting p38 MAPK	ND ^2^	[157]
Compound **36**	592.66	Directly inhibiting p38 MAPK	ND ^2^	[162]
Compound **37**	616.68	Directly inhibiting p38 MAPK	ND ^2^	[162]

^1^ Molecular Weight; ^2^ Not Determined.

## Abbreviations

The following abbreviations are used in this manuscript:

AD	Alzheimer’s disease
FDA	Food and Drug Administration
AchE	acetylcholinesterase
NMDAR	*N*-methyl-d-aspartate receptor
MAPK	mitogen-activated protein kinase
ROS	reactive oxygen species
JNK	*c*-jun N-terminal kinase
ERK	extracellular signal-regulated kinase
TNF-α	tumor necrosis factor-α
IL-1	interleukin-1
LPS	lipopolysaccharide
ATP	adenosine triphosphate
CNS	central nervous systems
Aβ	amyloid-β
NFTs	neurofibrillary tangles
APP	amyloid precursor protein
PS	presenilin
NO	nitric oxide
NSAIDs	nonsteroidal anti-inflammatory drugs
*i*NOS	inducible nitric oxide synthase
NF-kB	nuclear factor kappa-light-chain-enhancer of activated B cells
LTP	long-term potentiation
LTD	long-term depression
mGluR	metabotropic glutamate receptor
AMPAR	α-amino-3-hydroxy-5-methyl-4-isoxazolepropionic acid receptor
BBB	blood-brain barrier
